# India’s private sector engagement model improved TB notification and drug uptake

**DOI:** 10.5588/pha.26.0018

**Published:** 2026-08-24

**Authors:** S. Gupta, S.K. Mattoo, R. Rao, P. Manickam, K. Selvaraj, K. Soundappan, A.R. Singh, K. Mehta, S. Shanmugasundaram, U.B. Singh

**Affiliations:** 1 Karnataka Health Promotion Trust, New Delhi, India;; 2 Central TB Division, Ministry of Health and Family Welfare, New Delhi, India;; 3 Division of Health Systems Research, ICMR National Institute of Epidemiology, Chennai, Tamil Nadu;; 4 Department of Community and Family Medicine, AIIMS, Madurai, Tamil Nadu;; 5 Department of Community Medicine and School of Public Health, Post Graduate Institute of Medical Education and Research, Chandigarh, India;; 6 Dahdahleh Institute of Global Health Research, Faculty of Health, York University, Toronto, Canada;; 7 Department of Community Medicine, Government Medical College, Shahdol, Madhya Pradesh, India;; 8 Department of Community Medicine, GMERS Medical College, Gotri, Vadodara, Gujarat, India;; 9 Department of Community Medicine and Family Medicine, All India Institute of Medical Sciences, Hyderabad, India;; 10 Department of Microbiology, All India Institute of Medical Sciences, New Delhi, India.

**Keywords:** tuberculosis, NTEP, M-PHISOR, private sector engagement, public–private mix

## Abstract

**SETTINGS:**

Since 2018, India’s National Tuberculosis Elimination Programme has adopted a patient-provider support agency (PPSA) model to engage the private sector using domestic resources.

**OBJECTIVE:**

To evaluate the effectiveness of the PPSA model for private-sector TB notifications, care services, and treatment success in 42 districts across six Indian states and to explore enablers and challenges of PPSA implementation.

**DESIGN:**

A mixed-methods study. We performed interrupted time-series analysis using the segmental regression model to quantify level and monthly changes in private sector TB indicators before (2019–2021) and after PPSA implementation (2021–2024) and interviewed stakeholders regarding enablers and challenges of PPSA implementation.

**RESULTS:**

PPSA initiation significantly increased private-sector TB notification rates (immediate change) from 43.5 in 2019 to 72.7 per 100,000 in 2021 and sustained till 2024. Similarly, number of private facilities notifying TB increased from 1,040 to 1,858 and prescription of free fixed dose combination drugs by private providers increased from <1% to 14%. These gains were not accompanied by improvements in other TB care services and treatment success. Stakeholders offered insights and reasons for these variations.

**CONCLUSION:**

The PPSA model improved notification and drug uptake and will require strengthening of quality-of-care services for future scale-up.

India has the highest TB burden globally (estimated 2.7 million cases)^1^ and has made notable progress in reducing missed notifications from 1.2 million in 2015 to 0.3 million in 2023,^2,3^ with 2.5 million TB cases notified in 2023.^2^ Although India’s National Tuberculosis Elimination Programme (NTEP) is mostly delivered through the public sector, a large and heterogeneous private health care sector serves as a major first point of care for TB patients.^4–6^ In 2023, the private sector contributed 33% of overall TB notifications, highlighting its significant role in TB care.^2^ However, persistent challenges including underreporting, delays in diagnosis and treatment initiation, and suboptimal adherence to standards of TB care and treatment outcomes have been widely reported in this sector.^7–9^ To address these gaps and ensure universal access to TB care, the NTEP prioritised innovative approaches to engage private health care providers^8,10^ through donor-funded pilot projects. These include the patient-provider interface agency (PPIA) model in three cities (2013–2017) and the Global Fund–supported patient-provider support agency (PPSA) model across 105 cities (2018–2020).^11,12^

Small-scale studies of these projects documented increased TB notifications and reduced treatment delays.^11,12^ Building on this evidence, the NTEP adopted the PPSA model as a strategic approach for engaging the private sector,^10^ transitioned to domestic funding in 2018 and scaled implementation to 235 districts by 2024.^2^ However, national-level evidence on its effectiveness remained limited. Further, the previous studies mostly documented service level indicators, with limited attention to contextual factors shaping PPSA implementation and were restricted to smaller geographies.^13,14^

Recognising the need to generate national-level evidence, we previously compared district-level TB service indicators between PPSA and non-PPSA districts in 2023.^15^ We documented a 2.5-fold higher TB notification rate, 46% reduction in pretreatment loss to follow-up, and 50% reduction in treatment delays (>7 days) in PPSA districts. However, that analysis was limited by its cross-sectional design and inability to account for district-level characteristics such as demographics, private-sector availability, and baseline TB notification levels. It also did not assess changes before and after PPSA implementation or temporal variations in TB care indicators over time.

We have therefore evaluated the effectiveness of the PPSA model in districts where PPSA operations commenced in 2021, assessing private sector TB notification, care services, and treatment outcomes before and after PPSA implementation. Additionally, we explored stakeholders’ perspectives on enablers and challenges in PPSA implementation at State and District levels.

## METHODS

This was an explanatory sequential mixed-methods study. We quantified the effectiveness of the PPSA model through a quasi-experimental design followed by stakeholder interviews to explore enablers and challenges using a cross-sectional design.

### General setting

India’s NTEP operates in decentralised manner through Central TB Division, 36 State TB Cells, and 771 District TB Centres at national, state, and district levels, respectively. To strengthen private-sector TB case detection, TB notification to NTEP was made mandated for all private practitioners, laboratories, and pharmacies through a government Gazette notification (REGD. NO. D. L.-33004/99, 19 March 2018).^16^ Under NTEP, all diagnosed TB cases are reported in a web-based patient management system, *Ni-kshay*.^17^ Since 2018, NTEP has provided financial incentives to private providers to facilitate TB notification in *Ni-kshay*.^2^

### Specific setting

PPSA serves as an intermediary between NTEP and the private health sector to offer comprehensive TB services by engaging private providers and linking them with NTEP. PPSA supports private provider mapping, sensitisation on standard TB guidelines, TB notification in *Ni-kshay*, and improved patient care. It facilitates linkage of persons with TB (PwTB) to free diagnostics and drugs from public facilities; counselling and treatment adherence; HIV/diabetes screening; drug susceptibility testing; linkage to social benefit schemes such as *Ni-kshay poshan yojana* (NPY); and treatment outcome reporting.^18^ States and districts engage PPSAs based on their service delivery gaps, burden of private sector TB cases, and private-sector presence. Non-governmental organisations are contracted as PPSA on performance-based funding.^19^ In districts without PPSA, routine NTEP staff manages private-sector engagement. Using domestic funding, PPSA expanded from 15 districts in Maharashtra in 2018 to 135 districts across 12 states by 2021.

### Effectiveness of PPSA model

We considered 55 districts from 7 states (Andhra Pradesh [n = 14], Assam [n = 13], Chhattisgarh [n = 6], Goa [n = 2], Maharashtra [n = 11], Odisha [n = 7], and Punjab [n = 2]) where PPSA operations commenced in 2021. These districts were selected to examine temporal trends in TB care indicators before (2019–2021) and after PPSA implementation (2021–2024). However, due to unavailability of private sector notification data, 11 districts were excluded. The final analysis included 42 districts from 6 states representing most regions of India (see Supplementary Data File S1 and Supplementary Data Figure S1).

### Data abstraction

Monthly secondary programmatic data on private sector TB notifications and care services were retrieved from *Ni-kshay* for January 2019 to December 2024. PPSA effectiveness before and after implementation was assessed through change in the following indicators (definitions available in Supplementary Data File S2): 1) Private sector TB notification rate; 2) Pretreatment loss-to-follow-up (LFU) among notified private TB cases; 3) Delay in treatment initiation >3 days among TB cases initiated on treatment; 4) Public health action/TB care services including HIV testing, diabetes screening, rifampicin susceptibility through nucleic acid amplification test (NAAT), uptake of fixed drug combination (FDC) drugs, and bank details linkage for NPY benefit; and 5) Treatment success.

### Data analysis

Aggregate monthly data on private sector TB notification, pretreatment LFU, treatment delay, TB care services, and treatment outcomes were collated for 42 districts from January 2019 to December 2024. Each district contributed 72 time-points. District-level data were later aggregated to state and national levels. An interrupted time-series (ITS) analysis was performed using a segmented regression model to assess PPSA effectiveness. Time-series data included ‘sequential monthly outcome indicators’ from January 2019 to December 2024. The ‘PPSA intervention point’ was set at August 2021, corresponding to PPSA initiation in all 42 districts. The study period was classified into three different phases i) ‘January 2019 to July 2021’ referred as the ‘No-PPSA Phase’ or ‘baseline period’ including a 5-month preparatory period in some districts; ii) ‘August 2021 to February 2024’ considered as the ‘PPSA Phase’ during which all 42 districts had PPSA support; and iii) ‘March to December 2024’ considered as the ‘Partial PPSA Phase’ when 27 districts lacked functional PPSA due to delays in contract renewal. Immediate changes were estimated from level change while changes over time were assessed using regression coefficients for slope (trend) representing monthly changes. Post-intervention trends were also compared with baseline slopes. Stationarity of the series was checked to assess the need for autocorrelation adjustment. Data was analysed using STATA version 18 (StataCorp, 2023, College Station, TX, USA).

### Stakeholders’ perspectives on PPSA implementation

Following quantitative analysis, state TB officers (STOs), district TB officers (DTOs), and state-nodal representatives of PPSA were selected through deviant case sampling from 10 districts and corresponding states for key informant interviews (KIIs). Stakeholders with experience in PPSA implementation or oversight during 2021–2024 were included.

### Data collection

The principal investigator (SG), an NTEP consultant with no role in PPSA implementation, conducted online KIIs during January–February 2026 using English language interview guides (available in Supplementary Data File S3). Telephonic oral informed consent and permission for audio-visual recording were obtained before interview. Repeat interviews were conducted where clarity was required. Audio-recorded responses were stored anonymously with strict confidentiality.

### Data analysis

Interviews were transcribed verbatim in English on the same day of interview (SG). Descriptive content analysis with manual coding was performed. Kurt Lewin’s force field analysis framework was used to identify driving forces (enablers) and restraining force (challenges) in PPSA implementation.^20^ A codebook (see Supplementary Data File S4) was developed using inductive themes emerging from the data. Similar codes were grouped into broader categories (SG and KLS). Findings from ITS analysis were interpreted alongside themes from stakeholder interviews.

### Ethical statement

Ethics approval for study conduct, telephonic oral consent, and audio recording of stakeholder interviews was obtained from the Institutional Human Ethics Committee of Indian Council of Medical Research National Institute of Epidemiology, Chennai (NIE/IHEC/A/202506-24 dated 6 July 2025).

## RESULTS

### Effectiveness of PPSA model

Forty-two districts across six study states reported 343,263 private sector TB notification between January 2019 and December 2024, with overall notification rate of 60 per 100,000 population. These PwTB were predominantly aged 15–44 years (53%) and men (57%).

The majority were new cases (89%), while 55% had pulmonary TB (55%) and 41% had extra pulmonary TB. Two percent had drug-resistant TB. Overall, 27% of notified cases had bacteriological confirmation during 2019–2024 (Figure).

**FIGURE.**
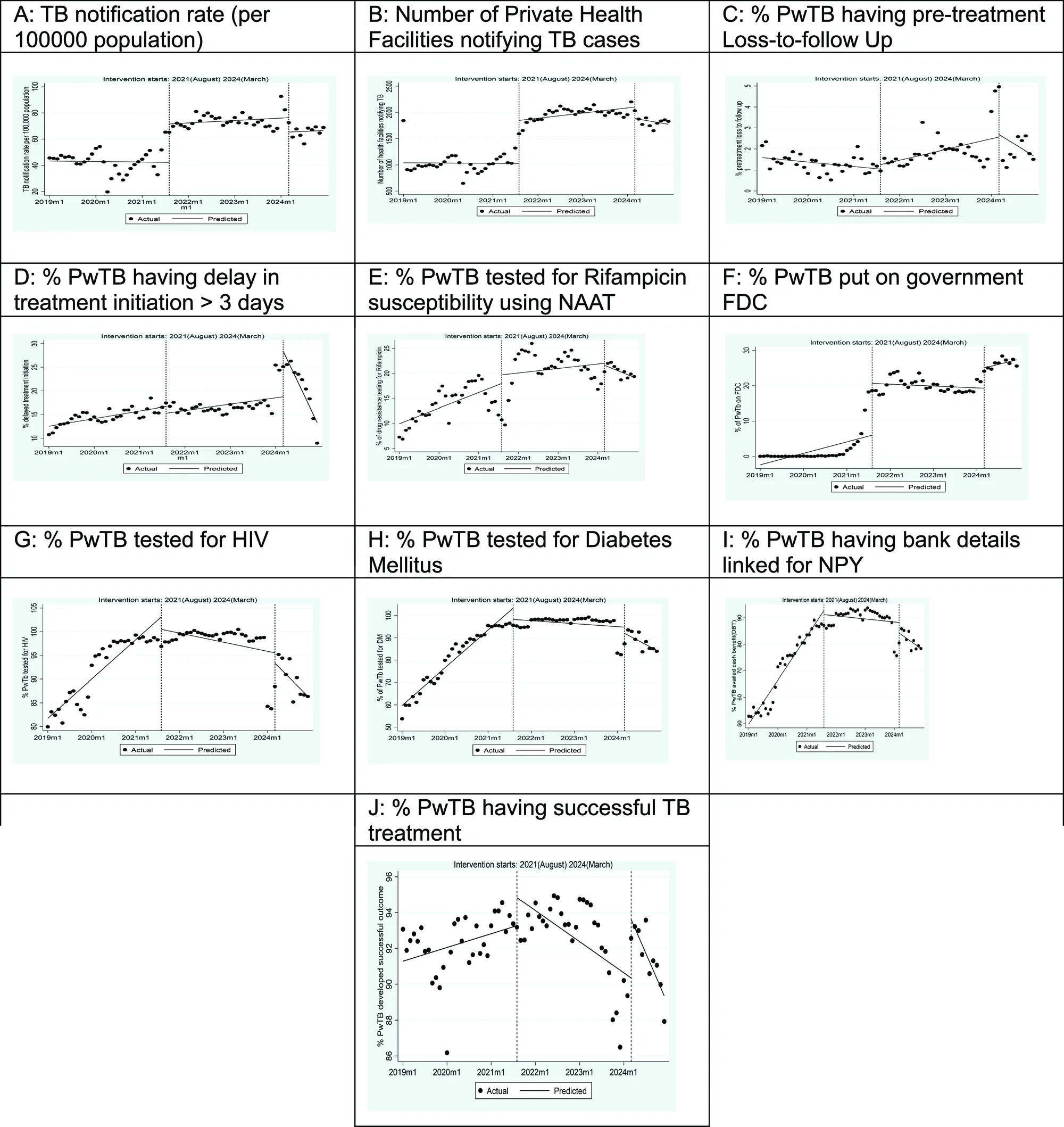
Monthly time trend distribution of Private sector TB notification, care services, and treatment success corresponding to the PPSA model during January 2019 to December 2024 in 42 districts across six states in India. **A)** TB notification rate (per 100,000 population); **B)** Number of private health facilities notifying TB cases; **C)** % PwTB having pretreatment loss-to-follow-up; **D)** % PwTB having delay in treatment initiation >3 days; **E)** % PwTB tested for rifampicin susceptibility using NAAT; **F)** % PwTB put on government FDC; **G)** % PwTB tested for HIV; **H)** % PwTB tested for diabetes mellitus; **I)** % PwTB having bank details linked for NPY; **J)** % PwTB having successful TB treatment. PwTB = persons with TB; PPSA = patient-provider support agency (interface agency to link private health facilities, their patients to NTEP for notification and quality free TB care); FDC = fixed dose combination; NAAT = nucleic acid amplification test; NPY = Ni-kshay Poshan Yojana.

### TB notification rate and frequency of notifying facilities

Following PPSA introduction, notification rate increased steeply from 43.5 per 100,000 in 2019 to 72.7 in 2021 and continued at the same level during the PPSA phase. During the partial PPSA phase, rates marginally dipped to 61.7 per 100,000 in 2024. This increase was accompanied by a significant rise in the number of private facilities notifying TB at PPSA initiation (1,858 vs. 1,040), which persisted throughout the PPSA phase. Conversely, the partial PPSA phase showed a significant reduction in number of notifying facilities (1,639 vs. 1,858). Details on ITS analysis of monthly change in private sector TB notification, care services, and treatment success are in the Supplementary Data Table S1.

### Pretreatment LFU

Following PPSA initiation, pretreatment LFU showed an increasing trend (β = 0.06%/month). However, compared with the baseline period, no major overall change was observed.

### Delay in treatment initiation (>3 days)

PPSA initiation did not result in an immediate reduction in treatment delay in the private sector (−1.4%, 95% confidence interval [CI]: −3.2 to 0.4). However, withdrawal of PPSA support led to a sudden and significant increase in delayed treatment initiation (9.7%, 95% CI: 5–14.3), followed by a subsequent decline (−1.8%, 95% CI: −2.5 to −1.1).

### TB care services

No significant changes were observed in rifampicin susceptibility testing through NAAT and testing for HIV and diabetes mellitus following PPSA implementation. A significant declining trend in microbiological confirmation of TB was observed after PPSA initiation (−0.6%, 95% CI: −0.8 to −0.4).

Uptake of programmatic FDC showed a consistent increasing trend during the PPSA phase (14.6 percentage-point change in 2021 compared to <1% in 2019) and during the partial PPSA phase (20.3 percentage-point change in 2024 compared to year 2021). Bank account linkage for NPY was already high (∼90%) at PPSA initiation and remained stable thereafter.

### Treatment success

A significant declining trend in successful treatment outcomes was observed during both the PPSA phase (−0.2%, 95% CI: −0.3 to −0.1) and the partial PPSA phase (−0.3%, 95% CI: −0.6 to −0.1).

### Stakeholders’ perspective on PPSA implementation

We included 13 KIIs (3 STOs, 6 DTOs, and 4 state PPSA nodal persons). Participants had a median (interquartile range) experience of 5 years (3,12) in TB programmes and 4.5 years (3,8) in PPSA implementation/oversight; two participants were women. We categorised findings into two categories: 1) Government health system/NTEP-related and 2) PPSA structure/services-related for ‘enablers’ and ‘challenges’ (see Supplementary Data Tables S2 and S3).

### Theme 1: perceived enablers in PPSA implementation

Stakeholders identified strong coordination with NTEP and regular performance review of PPSA as key facilitators. Other enablers included provision of free diagnostics and drugs to private-sector patients, trained PPSA staff, financial incentives for notification, and continued medical education for provider sensitisation.

### Theme 2: perceived challenges in PPSA implementation

Key challenges included interruptions in diagnostics and drugs, delayed fund release to PPSA, and selective prioritisation of payment linked indicators by PPSA. Stakeholders also highlighted inadequate field staff, limited staff capacity, patient migration, and confidentiality concerns as barriers to timely service delivery.

## DISCUSSION

Our study found consistent improvements in private-sector TB notifications, number of notifying private facilities, and uptake of programmatic FDC following PPSA implementation compared to baseline period. However, these gains were not accompanied by improvements in other TB care services and treatment outcomes.

The observed improvements in notification rate and notifying facilities is consistent with state-based studies reporting 18%–50% increase in private TB notification after PPSA implementation.^13,14^ Similar improvements in case reporting have also been documented in other disease control programmes^21^ and alternative provider engagement models.^22^

The significant increase in FDC uptake among private-sector PwTB implies strengthened mechanisms for supplying and recording public-sector drugs to private providers after PPSA introduction.^23^ Once institutionalised, these processes remained effective even during the partial PPSA period. Similar improvements in FDC uptake^15^ have been reported from previous PPSA studies when systematic drug supply chains were ensured.^12,24,25^

These findings differ from our previous study, where non-PPSA districts showed better FDC coverage.^15^ This may reflect state-level variation in availability of drugs across compared districts. The present design overcomes this variability and hence reflects true trends. In contrast, quality of care indicators showed limited improvement. Previous state-specific PPSA evaluation reported better outcomes,^13,14^ suggesting variation in resources, monitoring, and implementation priorities across states. Several factors may explain the limited gains observed in the present study. First, many of these services depended on patient follow-up, whereas incomplete contact information and patient migration to other districts limited PPSA’s tracking and quality services. Second, services such as NAAT relied on uninterrupted availability of consumables and laboratory personnel within health system and were vulnerable to supply-chain disruptions.^26^ Stakeholders also reflected these constraints in interviews. Third, studies suggest provider hesitancy in offering HIV testing due to patient reluctance, privacy and limited provider sensitisation in spite of programme recommendations.^27,28^ Fourth, many quality-of-care services carry lower payment weightage than notification and outcome in PPSA performance–based contracts,^19^ resulting in lower prioritisation by PPSAs. Stakeholders similarly reported selective operational focus on highly incentivised indicators. Finally, routine programmatic reviews generally focus on notification and provider mapping, with limited focus on diagnostic quality and comprehensive care indicators, reducing accountability of these services by PPSA. These gaps also explain the decline in treatment success over time.

The quasi-experimental design provided a feasible approach to assess the effectiveness of a government-funded private-sector engagement model while addressing limitations of our previous work. Using ITS analysis on the same cohort of districts adjusted for confounders such as population, health-system factors, TB burden, seasonality, and private health care volume by treating each district as its self-control. Qualitative findings provided pertinent insights to inform PPSA refinement for future scale-up.

The findings should be interpreted considering some limitations. First, PPSA rollout timelines varied across states depending on contractual agreement (Supplementary Data Table S1). To address potential misspecification trend bias, we performed sensitivity analysis considering the initial 6 months as a preparatory PPSA phase. As this did not change the trend, the three-phase model was retained for parsimony. Second, apart from the COVID-19 pandemic, no major concurrent interventions or interruptions could have influenced observed trends. While the pandemic may have some effect, the PPSA start point (August 2021) included a stable 15-month pre-COVID period and over 30 months of post-COVID data, supporting robustness of the findings. Finally, perspectives of private providers and patients were not explored in stakeholders’ interviews and should be examined in future research.

Notwithstanding the limitations, the findings can inform refinement and expansion of the PPSA model regions and guide adaptation for other public health priorities. Future research should evaluate the cost and financial sustainability of PPSA implementation under domestic financing, and its optimisation for future scale-up.

## CONCLUSION

In India, the domestically funded PPSA model effectively increased private-sector TB notification, provider engagement, and uptake of programmatic drugs. However, its impact on diagnostic quality, care services, and treatment success remained limited. Greater emphasis is needed on quality-oriented TB care services, stronger linkage of these indicators with performance-based financing, and comprehensive monitoring to improve PPSA effectiveness.

## Supplementary Material
