# Supplementary material for: India’s private sector engagement model improved TB notification and drug uptake
Source: Public Health Action. 2026 Aug 24;16(3):177–82. doi: 10.5588/pha.26.0018 (PMC13502710; doi:10.5588/pha.26.0018)
Supplement: Supplementary file 1 [file pha26-0018_supplementarydata1.pdf]

**Supplementary file 1: List of States and districts having initiation of Patient Provider Support Agency (PPSA) operation in 2021 and included in the study**

| State Name (n=6) | District Name (n=42) | PPSA operations start month in 2021 | PPSA operations continuation status in 2024 |
|---|---|---|---|
| Andhra Pradesh | Ananthapuramu | Aug 2021 | Operational till December 2024 |
| | East Godavari | Aug 2021 | Operational till December 2024 |
| | Guntur | Aug 2021 | Operational till December 2024 |
| | Krishna | Aug 2021 | Operational till December 2024 |
| | PRAKASAM | Aug 2021 | Operational till December 2024 |
| | Sri Potti Sriramulu Nellore | Aug 2021 | Operational till December 2024 |
| | Y.S.R | Aug 2021 | Operational till December 2024 |
| Assam | Barpeta | Feb 2021 | Operational till February 2024 |
| | Cachar | Feb 2021 | Operational till February 2024 |
| | Dibrugarh | Feb 2021 | Operational till February 2024 |
| | Goalpara | Feb 2021 | Operational till February 2024 |
| | Jorhat | Feb 2021 | Operational till February 2024 |
| | Kamrup Metro | Feb 2021 | Operational till February 2024 |
| | Nagaon | Feb 2021 | Operational till February 2024 |
| | Sibsagar | Feb 2021 | Operational till February 2024 |
| | Sonitpur | Feb 2021 | Operational till February 2024 |
| | Tinsukia | Feb 2021 | Operational till February 2024 |
| Chhattisgarh | Bilaspur | April 2021 | Operational till April 2024 |
| | Dhamtari | April 2021 | Operational till April 2024 |
| | Durg | April 2021 | Operational till April 2024 |
| | Korba | April 2021 | Operational till April 2024 |
| | Raigarh | April 2021 | Operational till April 2024 |
| | Raipur | April 2021 | Operational till April 2024 |
| Maharashtra | Dhule MC | June 2021 | Operational till December 2024 |
| | Jalgaon MC | June 2021 | Operational till December 2024 |
| | Latur | February 2021 | Operational till December 2024 |
| | Nanded Waghala MC | February 2021 | Operational till December 2024 |
| | Nandurbar | February 2021 | Operational till December 2024 |
| | Parbhani | February 2021 | Operational till December 2024 |
| | Akola MC | March 2021 | Till February 2024, restarted for September to December 2024 |

| | | | |
|---|---|---|---|
| | Amravati MC | February 2021 | Till February 2024, restarted for September to December 2024 |
| | Chandrapur | March 2021 | Till February 2024, restarted for September to December 2024 |
| | Nagpur MC | February 2021 | Till February 2024, restarted for September to December 2024 |
| | Pune Rural | March 2021 | Till February 2024, restarted for September to December 2024 |
| Odisha | Bhubaneshwar MC | July 2021 | Operational till March 2024 |
| | Cuttack | June 2021 | Operational till March 2024 |
| | Ganjam | June 2021 | Operational till March 2024 |
| | Sundargarh | September 2021 | Operational till March 2024 |
| | Khordha | July 2021 | Operational till March 2024 |
| | SAMBALPUR | June 2021 | Operational till March 2024 |
| Punjab | Amritsar | April 2021 | Operational till December 2024 |
| | Patiala | April 2021 | Operational till December 2024 |

**Supplementary-Figure S1: Map of districts having initiation of PPSA operations in 2021, Included in the study**

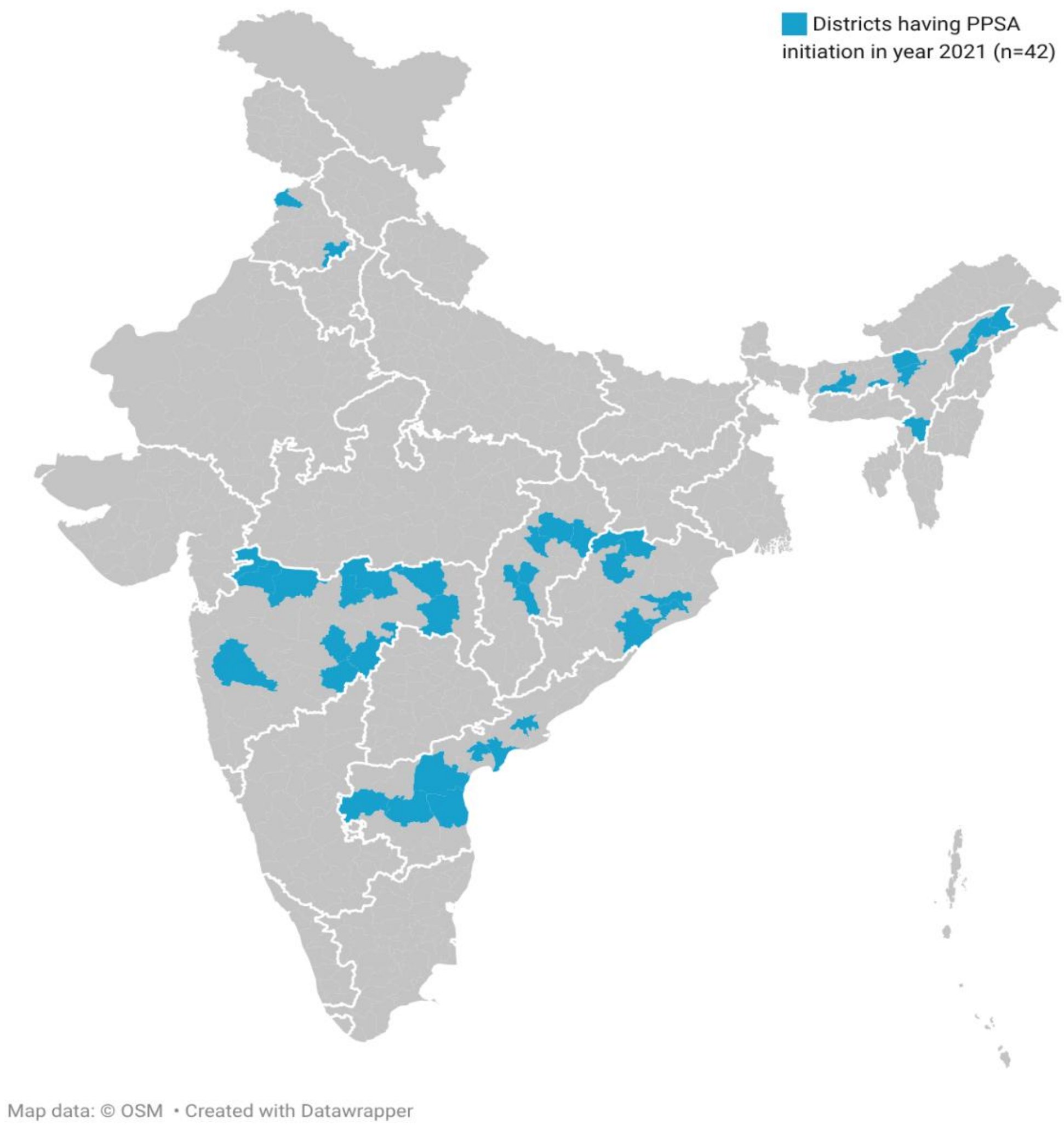

**Supplementary file 2: Operational Definitions**

1. *Private Provider/Private Health Care provider*: Any medical practitioner treating TB cases using modern medicine and is marked as a private provider/facility in *Ni-kshay* is considered a private provider.
2. *Patient Provider Support Agency (PPSA):* A PPSA refers to an agency/service provider usually a NGO contracted by the state or district for a defined period to deliver a package of services aimed at strengthening engagement with private sector healthcare providers. Its functions include- mapping and profiling private providers; facilitating the enrolment of presumptive TB patients and ensuring notification in Ni-kshay from private facilities; supporting linkages to free diagnostic and government drugs (FDC) services; providing patient counseling, adherence support, and comorbidity screening; coordinating universal drug susceptibility testing; assisting with Ni-kshay Poshan Yojana enrolment by submitting patient bank details to NTEP and conducting follow-up and reporting treatment outcomes. The scope and composition of services may vary across districts, depending on the specific terms of the contract.
3. *Commencement of PPSA operations*: The date of commencement of operations mentioned in the signed contract of PPSA will be taken as initiation of PPSA implementation in the district and will be used to assign before and after period for the analysis.
4. *End of PPSA operations: The end date of PPSA operations mentioned in the signed contract of PPSA, implying closure of activities supported by PPSA.*
5. *Pulmonary TB:* TB affecting the lung tissue. Any microbiological evidence of TB in the sputum or clinical TB in the lungs with or without the presence of the disease elsewhere is considered a case of pulmonary TB. Pulmonary TB marked in *Ni-kshay* will be taken as Pulmonary TB.
6. *Extra-pulmonary TB:* TB affecting any parts of the body other than the lungs is said to be extra-pulmonary TB. If a patient shows the presence of TB in the lungs along with sites other than the lungs, it will be considered a case of pulmonary TB. Extra-pulmonary TB marked in *Ni-kshay* will be taken as Extra-Pulmonary TB.

7. Drug-resistant TB (DR-TB): Microbiologically-confirmed TB patients with evidence of resistance to one or more conventionally used anti-TB drugs. In this paper, DR-TB is used for TB patients marked as DRTB in the *Ni-kshay* notification register.
8. *Linked with Programmatic FDC:* Notified private sector TB patients who were linked to and provided programmatic fixed drug combination (FDC) while still on consultation/treatment with a private doctor.
9. *Ni-kshay Poshan Yojana (NPY) and linkage to NPY*: NTEP offers a direct benefit transfer incentive to support the nutrition of TB patients (Rs. 500 per month for the entire treatment duration. Every notified TB patient (both public and private) is eligible for this. When the bank account detail of a patient is available in *Ni-kshay* and is seeded will be considered as linked for NPY.
10. *Pretreatment loss to follow up*: Gap of number of notified TB cases and number of TB cases initiated on treatment as per *Ni-kshay* in within a set timeframe for a district/state will be counted as pretreatment loss to follow up.
11. *Pre-treatment delay*: The NTEP program focuses on the start of TB treatment within 7 days of diagnosis. A delay of more than 7 days between the date of diagnosis and the date of treatment initiation will be considered a pre-treatment delay.
12. *Nucleic Acid Amplification Technology (NAAT*): Molecular Cartridge-Based Nucleic Acid Amplification Technology using Computerised GeneXpert/Truenat Machines. This technology detects Mycobacterium TB along with the rifampicin resistance status.
13. *Valid Drug susceptibility test:* Results from WHO recommended rapid molecular test indicating susceptibility as resistance detected or resistance not detected. It excluded all other results such as error, invalid and indeterminate.
14. *Microbiologically confirmed TB:* TB diagnosed in a biological specimen by smear microscopy, culture, or a WHO-endorsed rapid molecular test adopted by NTEP
15. *Upfront NAAT:* When NAAT test is offered as first test to diagnose presumptive TB sample, it is termed upfront NAAT, which detect TB mycobacteria as well as susceptibility to TB drugs.

16. *Private sector TB notification rate:* It is the number of TB cases diagnosed and notified by private sector providers in a given year, per 100,000 population.
17. *Pretreatment loss-to follow-up (LFU) among notified private TB cases:* The notified TB cases which were reported loss-to -follow in Nik-shay before initiation of their TB treatment.
18. *Delay in treatment initiation >than 3 days among TB cases initiated on treatment:* The TB cases which were initiated on TB treatment after three days from their date of diagnosis in Ni-kshay out of the total cases initiated on treatment. For the study time taken in initiating treatment for more than 3 days from diagnosis has been considered as delay.
19. Public health action/TB care services -Human Immunodeficiency Virus (HIV), Diabetes, rifampicin susceptibility testing through Nucleic acid amplification test (NAAT) testing, uptake of Fixed Drug Combination (FDC) drugs, bank details linkage for Nikshay Poshan Yojana (NPY) benefit
20. *TB care services*: A set of services which are to be offered to all notified TB cases (both in public and private sector) as part of public health action till treatment completion as per NTEP program guidelines. For the study TB care services include:
    a. Offering Nucleic Acid Amplification test (NAAT) for drug susceptibility testing (DST) to all the notified TB cases
    b. Linkage to programmatic fixed drug combination (FDC), among cases initiated on TB treatment
    c. Co-morbidity testing (HIV and diabetes mellitus), among cases initiated on TB treatment
    d. Bank detail linkage for Nikshay Poshan Yojana among cases initiated on TB treatment
21. *Treatment success:* It is the sum of all TB patients who are reported as “cured” and as “treatment completed” in Nikshay. For the study, treatment success among TB patient initiated on treatment has been considered.

**Supplementary file 3 (i): Interview Guide for Key Informant Interview (NTEP official-State/District)**

Topic: To explore the stakeholder's perspective towards enablers and barriers influencing the implementation of the PPSA model under NTEP

Name of the Participant____________

Current Designation/Organization______________

Date of Interview:______________

Interview start and end time:___

Mode of Interview : virtual video call

Purpose:

This study is being conducted to evaluate the effectiveness of PPSA model in improving private sector TB care services in districts where PPSA operations commenced during 2021 in India. We intends to explore stakeholders perspective towards enablers and barriers influencing the implementation of the PPSA model. Key stakeholder interviews (KII) of State TB officer, District TB Officer, State and district nodal person of PPSA from selected states/districts where PPSA operations commenced in 2021 will be conducted. Stakeholders who have worked in these 44 districts among 6 states from 2021 to 2024 are being interviewed. Ethical clearance for the study has been obtained from ICMR-NIE human ethics committee.

I will briefly explain the details of the study using participant information sheet. I am thankful to you for showing your willingness and interest for this interview. I would like to inform you that it will be 30-45 minutes long interview. In case, you don't want to respond to a question, you are free to skip the question. We would like to seek your informed consent (written) for conducting this interview. Also, we would like to seek your permission and consent to audio record the interview for analysis purposes.

**Interview Question's:**

At the outset, I would like to learn some preliminary questions about your:

i. Designation (between 2021-2024)/Organization______________
ii. Years of Experience of working as STO/DTO ______________
iii. Years of Experience of overseeing PPSA ______________

1. In your opinion, How was the private sector TB performance in your state/district between 2021-2024 with respect to:
    a. Notification,

    b. Public health actions (PHA)
    c. Treatment success
2. What do you know about PPSA and its functions?
3. What are your views about role/contribution of PPSA in private sector performance of state/district between 2021-2024 with respect to:
    a. Notification,
    b. Public health actions (PHA)
    c. Treatment success
4. What were the enablers/facilitators for PPSA implementation in your opinion /perception between 2021-24 with respect to:
    a. Contracting PPSA
    b. Delivery of services by PPSA
    c. Achievement of assigned targets
        i. For notification
        ii. Public health actions (PHA)
        iii. Treatment success
5. What were the barriers/challenges for PPSA implementation in your opinion /perception between 2021-24 with respect to:
    a. Contracting PPSA
    b. Delivery of services by PPSA
    c. Achievement of assigned targets
        i. For notification
        ii. Public health actions (PHA)
        iii. Treatment success
6. How the mentioned challenges can be mitigated to facilitate better functioning of PPSA?
7. Is there anything else that you wish to convey or mention?

Thank you for your time and active participation!

## Supplementary file 3 (ii): Interview Guide for Key Informant Interview (PPSA staff)

Topic: To explore the stakeholder's perspective towards enablers and barriers influencing the implementation of the PPSA model under NTEP

Name of the Participant____________

Current Designation/Organization______________

Date of Interview:______________

Interview start and end time:___

Mode of Interview : virtual video call

Purpose:

This study is being conducted to evaluate the effectiveness of PPSA model in improving private sector TB care services in districts where PPSA operations commenced during 2021 in India. We intends to explore stakeholders perspective towards enablers and barriers influencing the implementation of the PPSA model. Key stakeholder interviews (KII) of State TB officer, District TB Officer, State and district nodal person of PPSA from selected states/districts where PPSA operations commenced in 2021 will be conducted. Stakeholders who have worked in these 44 districts among 6 states from 2021 to 2024 are being interviewed. Ethical clearance for the study has been obtained from ICMR-NIE human ethics committee.

I will briefly explain the details of the study using participant information sheet. I am thankful to you for showing your willingness and interest for this interview. I would like to inform you that it will be 30-45 minutes long interview. In case, you don't want to respond to a question, you are free to skip the question. We would like to seek your informed consent (written) for conducting this interview. Also, we would like to seek your permission and consent to audio record the interview for analysis purposes.

**Interview Questions:**

At the outset, I would like to learn some preliminary questions about your:

iv. Designation (between 2021-2024)/Organization______________
v. Years of Experience of working with PPSA (past as well current)___

8. How was the private sector TB performance in your state/district between 2021-2024 with respect to:
    a. Notification,
    b. Public health actions (PHA)
    c. Treatment success
9. What do you know about PPSA and its functions?

10. What are your views about role/contribution of PPSA in private sector performance of state/district between 2021-2024 with respect to:
    a. Notification,
    b. Public health actions (PHA)
    c. Treatment success
11. What were the enablers/facilitators for PPSA implementation in your opinion /perception between 2021-24 with respect to:
    a. Delivery of services by PPSA
    b. Achievement of assigned targets
        i. For notification
        ii. Public health actions (PHA)
        iii. Treatment success
12. What were the barriers/challenges for PPSA implementation in your opinion /perception between 2021-24 with respect to:
    a. Delivery of services by PPSA
    b. Achievement of assigned targets
        i. For notification
        ii. Public health actions (PHA)
        iii. Treatment success
13. How the mentioned challenges can be mitigated to facilitate better functioning of PPSA?
14. Is there anything that you wish to convey or mention ?

    Thank you for your time and active participation!

**Supplementary file 4 (a): Codebook of perceived Enablers by key stakeholder for PPSA implementation**

| Transcript No. | | PPSA staff 1 | PPSA staff 2 | PPSA staff 3 | PPSA staff 4 | DTO1 | DTO2 | DTO3 | DTO4 | DTO5 | DTO6 | STO1 | STO2 | STO3 |
|---|---|---|---|---|---|---|---|---|---|---|---|---|---|---|
| **Codes (Category/subcategory wise)** | **Frequency of codes** | | | | | | | | | | | | | |
| **Government health system and NTEP related** | | | | | | | | | | | | | | |
| Coordination and liasoning support from NTEP | 7 | | 1 | 1 | | | | 1 | | 1 | 1 | | 1 | 1 |
| Regular reviews and performance monitoring of PPSA | 11 | 1 | | 1 | | 1 | 1 | 1 | 1 | 1 | 1 | 1 | 1 | 1 |
| Providing free NAAT testing and FDC drugs under NTEP | 5 | 1 | | 1 | | 1 | 1 | | | | | | | 1 |
| Performance linked payment to PPSA | 7 | 1 | 1 | | | | 1 | | 1 | | 1 | | 1 | 1 |
| **PPSA structure and services related** | | | | | | | | | | | | | | |
| Awareness generation for private providers | 4 | | | 1 | | | | 1 | | | 1 | | | 1 |
| Trained and experienced PPSA staff | 4 | | | 1 | | | | 1 | 1 | | | | | 1 |
| Innovative intervention by PPSA | 4 | | | 1 | | | 1 | 1 | | | 1 | | | |
| Financial Incentives to private providers | 4 | 1 | | 1 | 1 | | 1 | | | | | | | |

**Supplementary file 4 (b): Codebook of perceived Challenges by key stakeholder for PPSA implementation**

| Transcript No. | | PPSA staff 1 | PPSA staff 2 | PPSA staff 3 | PPSA staff 4 | DTO1 | DTO2 | DTO3 | DTO4 | DTO5 | DTO6 | STO1 | STO2 | STO3 |
|---|---|---|---|---|---|---|---|---|---|---|---|---|---|---|
| **Codes (Category/subcategory wise)** | **Frequency of codes** | | | | | | | | | | | | | |
| **Government health system and NTEP related** | | | | | | | | | | | | | | |
| Interrupted diagnostic and drug supplies under NTEP | 6 | 1 | 1 | | | | | 1 | 1 | 1 | | | | 1 |
| Delayed fund release process under NTEP | 5 | 1 | | | | 1 | | 1 | 1 | | | | 1 | |
| Competitive priorities under NTEP stretching existing diagnostic resources | 2 | | 1 | | | | | | 1 | | | | | |
| **PPSA structure and services related** | | | | | | | | | | | | | | |
| Inadequate field staff and limited outreach support from PPSA | 3 | | | | | 1 | | | | 1 | | 1 | | |
| Suboptimal staff quality | 1 | | | | | | | | | 1 | | | | |
| Prioritization of selective payment indicators | 2 | | | 1 | | | | | | | | | 1 | |
| Issues related patient migration | 5 | | 1 | 1 | | 1 | | 1 | | | 1 | | | |
| Hesitancy in patients to avail services | 5 | | | 1 | 1 | 1 | | | | 1 | | 1 | | |

**Supplementary Result -Table 1: Interrupted time-series analysis of monthly change in private sector TB notification, care services and treatment success corresponding to Patient Provider Support Agency model (PPSA) operations during period January 2019 -Dec 2024 in 42 districts across six states, India**

| Outcome Indicators | No PPSA phase (January 2019 to July 2021) | PPSA phase (August 2021 to February 2024) | | Partial phase (March to December 2024) | |
|---|---|---|---|---|---|
| | Baseline level | Level change over baseline | Trend | Level change over PPSA phase | Trend |
| **TB notification rate (per 100,000 population)** | 43.5 (38.4 to 48.5) | 29.2* (18.8 to 39.6) | 0.2 (-0.3 to 0.7) | -11 * (-18.7 to -3.3) | -0.03 (-0.9 to 0.8) |
| *Private HF notifying TB cases (N)* | 1040 (831.9 to 1248.6) | 817.6 * (606.3-1028.9) | 8.6 (-3.6 to 20.9) | -219.3 * (-365.8 to -72.6) | -20.4 (-42.9 to 2.1) |
| **Pretreatment LFU (%)** | 1.6 (1.3 to1.9) | 0.2 (-0.4 to 0.8) | 0.06 * (0.01 to 0.1) | 0.1 (-1.6 to 1.9) | -0.2 (-0.4 to 0.1) |
| **Delay in treatment initiation > 3 days (%)** | 12.6 (11.6 to 13.6) | -1.4 (-3.2 to 0.4) | -0.02 (-0.2 to 0.1) | 9.7* (5 to 14. 3) | -1.8 * (-2.5 to -1.1) |
| **TB Care Services (%)** | | | | | |
| *Microbiologically confirmed TB cases* | 18.1 (16 to 20.2) | -0.21 (-3.6 to 3.2) | -0.6* (-0.8 to -0.4) | 0.5 (-2.4 to 3.3) | -0.16 (-0.5 to 0.2) |
| *Rifampicin susceptibility testing through NAAT done* | 9.9 (7.8 to 12.1) | 1.7 (-4.2 to 7.6) | -0.2 (-0.5 to 0.1) | -0.4 (-4.1 to 3.3) | -0.3 * (-0.7 to -0.01) |
| *HIV testing done* | 81.7 (79.6 to 83.9) | -2.6 (-6.3 to 1.1) | -0.9 * (-1.1 to -0.6) | -2.9 (-7.9 to 3.5) | -0.6 (-1.3 to 0.1) |
| *DM testing done* | 59.7 (56.9 to 62.5) | -5.1 * (-9.5 to -0.7) | -1.5 * (-1.8 to -1.2) | -2.8 (-8.6 to 2.9) | -0.7 * (-1.4 to -0.1) |
| *Initiated on government FDC* | -2.4 (-4.9 to 0.12) | 14.6 * (9.0 to 20.2) | -0.3 * (-0.6 to -0.1) | 5.7* (3.8 to 7.7) | 0.3 * (0.1 to 0.6) |
| *Bank detail linkage for NPY* | 49.8 (46.7 to 53.0) | -1.5 (-5.9 to 2.8) | -1.5 * (-1.8 to -1.2) | -4.0 (-9.8 to 1.8) | -0.6 (-1.1 to 0.03) |
| **Successful treatment (%)** | 91.3 (90 to 92.6) | 1.6 (-0.1 to 3.2) | -0.2* (-0.3 to -0.1) | 3.3 * (1.6 to 5.5) | -0.3 * (-0.6 to -0.1) |

*Data are β-coefficients (95% CIs) from the linear regression model. (P)- proportion of TB cases; (*) data points with statistically significant p value. PPSA- Patient provider support agency (interface agency to link private health facilities, their patients to NTEP for notification and quality free TB care, HF- Health facilities, LFU-Loss-to-follow up, FDC- Fixed dose combination; TB – tuberculosis; HIV – human immunodeficiency virus; DM- Diabetes Mellitus, NPY -Ni-kshay Poshan Yojana; NAAT-Nucleic acid amplification test*

**Supplementary Result -Table 2: Perceived enablers from key stakeholders' perspective influencing implementation of Patient Provider Support Agency intervention (PPSA) model across districts where the model initiated during year 2021, India**

| Categories | Subcategories | Verbatim Quotes |
|---|---|---|
| Government health system and NTEP related | Coordination and liasoning support from NTEP | "Gold standard is cooperation, coordination, and collaboration between NTEP and PPSA. Their (PPSA) smooth functioning depends upon it" [DTO 6, male] |
| | Regular reviews and performance monitoring of PPSA | "We conduct regular reviews. It is very essential to track their performance" [DTO 1, male] |
| | Providing free NAAT testing and FDC drugs under NTEP | "They (PPSA) can get free of cost NAAT test through programme with timely sharing of reports with private providers. That kind of helps them."[STO 3 male]<br><br>"Providing free of cost FDC was one of the major enablers" [PPSA staff 2, male] |
| | Performance linked payment to PPSA | "As their performance indicators are linked to their payments …it ensures accountability of PPSA towards those activities" [STO 2, male] |
| PPSA structure and services related | Awareness generation for private providers | "Continued Medical Education (CME)s have improved provider engagements and supported in improving active provider numbers." [DTO 6, male] |
| | Innovative intervention by PPSA | "Compounder incentive..worked in our model...They used to keep record like prescriptions and shared with our staff (PPSA). This was helpful because we got lot of cases due to this intervention" [PPSA staff 4, male] |
| | Financial Incentives to private providers | "Money is always a motivation. Incentives to PPs, pharmacist and laboratories has worked in improving notifications". [PPSA staff 4, male] |
| | Trained and experienced PPSA staff | "Having good staff with good training that is crucial for success of PPSA work" [DTO 4, male] |

*NTEP- National TB elimination programme; STO- State TB Officer, DTO-District TB Officer; NAAT- Nucleic acid amplification test; FDC- Fixed dose combination*

**Supplementary Result -Table 3: Perceived challenges from key stakeholders' perspective influencing implementation of Patient Provider Support Agency intervention (PPSA) model across districts where the model initiated during year 2021, India**

| Categories | Subcategories | Verbatim Quotes |
|---|---|---|
| **Government health system and NTEP related** | Interrupted diagnostic and drug supplies under NTEP | "When there is a shortage of the cartages, diagnosis…. its reporting cannot be done timely …., so the trust and rapport get break between PPSA staff and private providers" [PPSA staff 2, male] |
| | Delayed fund release process under NTEP | "We have not been able to pay our PPSA for last one complete year due to shortage of funds. ….it is a challenge. Their (PPSA) visits have been limited to absolute essential'[DTO 3, male] |
| | Competitive priorities under NTEP stretching existing diagnostic resources | "In ACF campaigns under TB Mukt Bharat abhiyaan, public sector sample load get so much that doing private sector sample testing NAAT becomes challenging" [DTO 4, male] |
| **PPSA structure and services related** | Inadequate field staff and limited outreach support from PPSA | "Private providers are more in number, while the PPSA staff provided for a district remains handful....They can't cover all these institutions and offer adequate services" [DTO 5, Female] |
| | Suboptimal staff quality | "knowledge levels of their (PPSA) people is not adequate…..they convey incomplete messages to the patients, not exactly what program is asking….." [DTO 5, Female] |
| | Prioritization of selective payment indicators | "They (PPSA) have selected payment linked indicators so they focus on notifications as it is linked to payment but TPT, differentiated TB care and presumptive case identification are not prioritized by them" [STO 2, male] |
| | Issues related patient migration | "Patients come from far off places and also lots of migrants are there…., who after initiating TB treatment used to go back to their native places. For many such patients, tracking used to get difficult" [PPSA staff 4, male] |
| | Hesitancy in patients to avail services | "Those patients who are from good economic background hesitate to give their bank account number and do not want home visit by the PPSA for fear of stigma…...it is a challenge for them as without phone numbers it gets difficult for them to track patients and follow up with them" [DTO 1, male] |

*NTEP- National TB elimination programme; STO- State TB Officer, DTO-District TB Officer; NAAT- Nucleic acid amplification test; FDC- Fixed dose combination*
